# Application of electric potential improves ethanol production from xylose by active sludge

**DOI:** 10.1186/s13068-021-02065-y

**Published:** 2021-11-17

**Authors:** Lei Chen, Mingpeng Wang, Zhaojie Zhang, Yujie Feng

**Affiliations:** 1 School of Life Science, Qufu Normal University, Qufu, 273165 People’s Republic of China; 2 State Key Laboratory of Urban Water Resource and Environment, Harbin Institute of Technology, Harbin, 150090 People’s Republic of China; 3 Department of Zoology and Physiology, University of Wyoming, Laramie, WY 82071 USA

**Keywords:** Xylose consumption, Ethanol production, Electro-fermentation, Extracellular electron transfer, Microbial community structure

## Abstract

**Background:**

Low-cost raw materials such as lignocellulosic materials have been utilized in second-generation ethanol production process. However, the sequential and slow conversion of xylose into target products remains one of the main challenges for realizing efficient industrial lignocellulosic biorefinery.

**Results:**

By applying different constant potentials to different microbial electrolysis cells with xylose as the sole carbon source, we analyzed the output of metabolites, microbial community structures, electron flow, and carbon flow in the process of xylose electro-fermentation by domesticated activated sludge. The bioreactors produced currents when applying positive potentials. The peak currents of the + 0.242 V, + 0.542 V and + 0.842 V reactors were 0.96 × 10^–6^ A, 3.36 × 10^–6^ A and 6.43 × 10^–6^ A, respectively. The application of potentials promoted the xylose consumption, and the maximum consumption rate in the + 0.542 V reactor was 95.5%, which was 34.8 times that of the reactor without applied potential. The potential application also promoted the production of ethanol and acetate. The maximum ethanol yield (0.652 mol mol^−1^ xylose) was obtained in the + 0.842 V reactor. The maximum acetate concentration (1,874 µmol L^−1^) was observed in the + 0.842 V reactor. The optimal potential for ethanol production was + 0.842 V with the maximum ethanol yield and energy saving. The application of positive potential caused the microorganisms to carry out ethanol fermentation, and the application of negative potential forced the microorganisms to carry out acetic fermentation. The potential application changed the diversity and community structure of microorganisms in the reactors, and the two most significantly changed families were *Paenibacillaceae* and *Bacillaceae*.

**Conclusion:**

The constructed microbial electrolysis cells with different potentials obtained better production yield and selectivity compared with the reactor without applied potential. Our work provides strategies for the subsequent fermentation processes with different needs.

**Supplementary Information:**

The online version contains supplementary material available at 10.1186/s13068-021-02065-y.

## Background

More than 80% of our present energy supply comes from fossil fuel resources. Increasing concern over the impact of these nonrenewable resources on climate change, human health, and ecosystems has prompted researchers to find renewable alternatives to meet our growing energy demand [1].

Ethanol is the most produced biofuel in the world. Traditionally, it is made from a large variety of carbohydrates (sugar cane, corn, sweet potato starch, etc.) [2, 3]. The second-generation ethanol production process utilizes low-cost raw materials such as lignocellulosic materials (sucrose, bagasse, corn stover, and straw). Lignocellulosic materials are mainly composed of cellulose (40–50%), hemicellulose (25–30%) and lignin (10–20%) [4]. Cellulose contains glucose, which can be effectively fermented into ethanol by *Saccharomyces cerevisiae*. However, the xylose in hemicellulose cannot be fermented by *Saccharomyces cerevisiae*, and its content sometimes accounts for 25% of the lignocellulosic material [5, 6]. Efficient conversion of xylose presented in lignocellulosic biomass is essential for the production of second-generation ethanol.

Biological conversion such as anaerobic digestion that converts wet biomass waste into bioethanol is a well-established technology. However, conversion of biomass waste to liquid fuels such as ethanol is only in the exploratory research phase [7]. For the biotransformation of most types of impure strains or substrates, it is difficult to control the microbial population and redox conditions, resulting in poor product selectivity and process stability [8]. The applied electric field can affect the fermentation environment and metabolic pathways of microbial cells through reduction or oxidation [9]. Through the use of electrodes, the electrical stimulation of microbial metabolism can control and optimize the fermentation environment to improve product yield and purity, and conducive to the growth of microbial cells [10].

In this study, the feasibility of using xylose and domesticated activated sludge to produce ethanol under different applied potentials was studied. By calculating the transferred electron equivalent, carbon conversion and energy conversion efficiency, the metabolic patterns of microorganisms under different potentials were analyzed. The optimal potentials for different purposes were evaluated, and the correlation between by-product production and microorganisms was discussed. Our study provides strategies for the further conversion and utilization of xylose.

## Results

### Enrichment of electroactive microorganisms that can utilize xylose

**Fig. 1:**
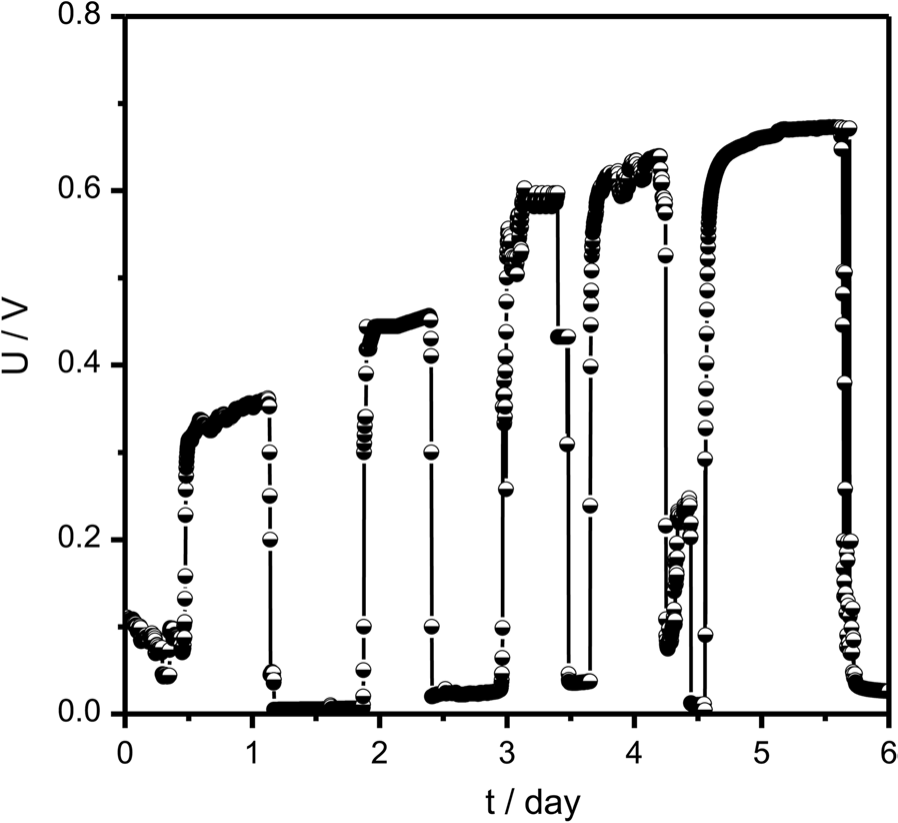
Voltage response of MFC to domestication. Voltage generation in an MFC with an external load of 1000 Ω

Xylose is one of the major components of fermentable sugars produced in straw. To enrich microorganisms which could produce electricity from xylose, microorganisms in sludge were acclimated with 4 g L^−1^ xylose in the air-cathode reactor. Xylose 4 g L^−1^ was inoculated into the anode chamber of the reactor, and the cell voltages were recorded over 6 days. The peak voltages rose gradually (Fig. 1). In the first cycle, the activated sludge generated a voltage of ~ 0.354 V. After the reactors running with xylose addition for five cycles, the peak voltage reached 0.672 V, which was 1.90 times higher than that of the first cycle.

### Current production from the sludge in a three-anode system fed with xylose

**Fig. 2:**
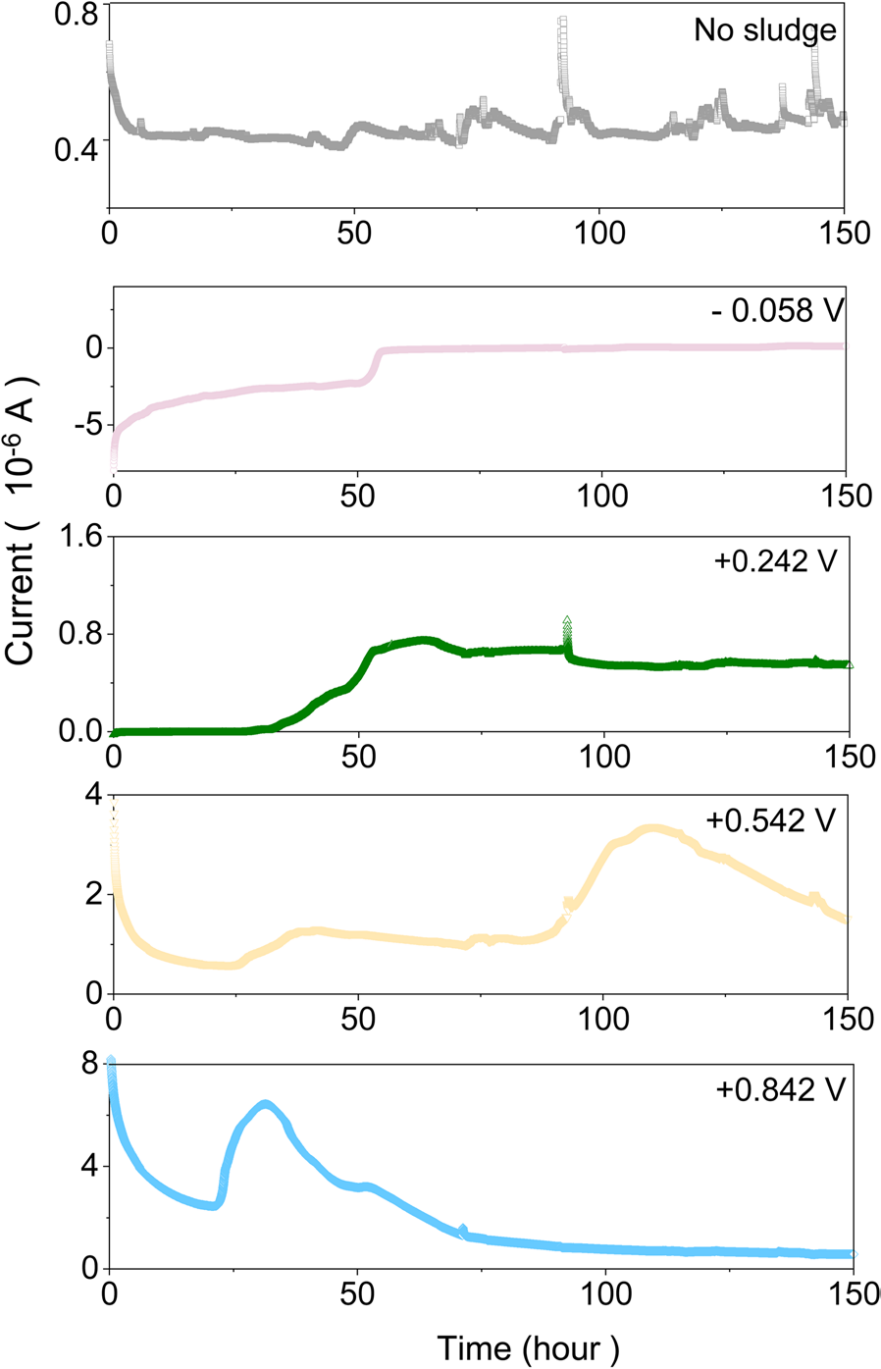
Current profile produced by the domesticated active sludge with applied potentials of − 0.058 V, + 0.242 V, + 0.542 V, and + 0.842 V vs. the standard hydrogen electrode

We used the MEC to accurately measure the electroactivity of the domesticated sludge with xylose over 150 h. Figure 2 showed the chronoamperometry results for different potentials against the standard hydrogen electrode. Sludge was the inoculum for the MEC. The reactor current was measured over 150 h. No current peak was observed for the − 0.058 V reactor or control reactor (without sludge inoculation).

In the + 0.242 V reactor, a current peak of 0.96 × 10^–6^ A appeared at 65 h. In the + 0.542 V reactor, a current peak of 3.36 × 10^–6^ A showed at 110 h. Bioreactors under a higher applied potential (+ 0.842 V) produced a higher current peak of 6.43 × 10^–6^ A at 31.5 h. This indicated that the microorganisms in sludge had electrogenic properties with xylose as the substrate and could transfer extracellular electrons to the electrodes. Application of a higher potential made the formation of electroactive biofilms easier, and the extracellular electron transfer (EET) between microorganisms was strengthened.

### Xylose consumption efficiency and production of byproducts

**Fig. 3:**
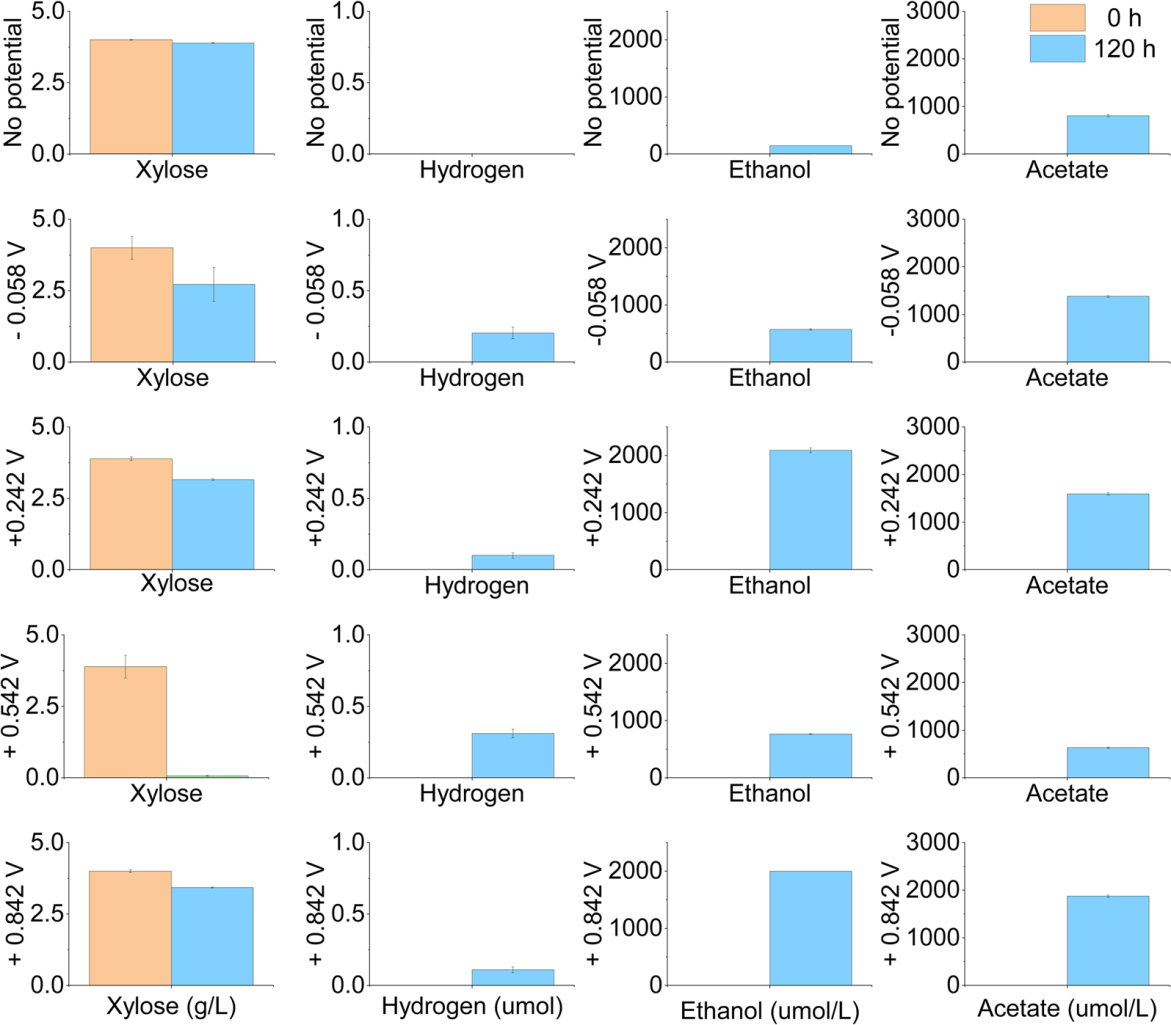
Xylose consumption and production of byproducts from the mixed culture at applied potentials of − 0.058 V, + 0.242 V, + 0.542 V, and + 0.842 V vs. the standard hydrogen electrode

Ethanol was the main product of xylose fermentation, followed by acetate and hydrogen. Figure 3 showed that xylose was used to a greater or lesser extent at 150 h in all reactors except for the one without the applied potential. The xylose consumption rates were below 100% in all reactors. The xylose consumption rate reached 2.75% in the reactor without potential application, 29.3% in the − 0.058 V reactor, 18.4% in the + 0.242 V reactor, 95.5% in the + 0.542 V reactor and 11.5% in the + 0.842 V reactor. The consumption rates of xylose in the reactors with potentials were significantly higher than that in the reactor with no applied potential. Among them, the highest consumption rate was 92.4% in the + 0.542 V reactor.

Ethanol production was improved after potential application. The lowest ethanol production (148 µmol L^−1^) was achieved in the reactor with no applied potential. The ethanol concentrations of the − 0.058 V, + 0.242 V, + 0.542 V, and + 0.842 V reactors were 567 µmol L^−1^, 2,093 µmol L^−1^, 764 µmol L^−1^, and 2,000 µmol L^−1^, respectively. The ethanol yield was 0.202 mol mol^−1^ xylose in the reactor with no applied potential. The ethanol yield of the − 0.058 V, + 0.242 V, + 0.542 V, and + 0.842 V reactors were 0.073, 0.426, 0.030, and 0.652 mol mol^−1^ xylose, respectively. The maximum ethanol yield was obtained in + 0.842 V reactor.

Acetate production was improved after potential application except the + 0.542 V reactor. The highest acetate concentration (1874 µmol L^−1^) was in the + 0.842 V reactor. In the reactor with no applied potential, the acetate concentration was only 805 µmol L^−1^. Acetate concentration in the − 0.058 V reactor was 1380 µmol L^−1^. Acetate concentration in the + 0.242 V reactor was 1594 µmol L^−1^.The minimum acetate concentration (632 µmol L^−1^) was obtained in the + 0.542 V reactor.

Hydrogen production was detected in reactors with applied potentials of − 0.058 V to + 0.842 V. No hydrogen was produced in the reactor with no applied potential. After applying potentials, 0.20, 0.12, 0.36 and 0.11 µmol hydrogen were produced from 4 g L^−1^ of xylose in the − 0.058 V, + 0.242 V, + 0.542 V, and + 0.842 V reactors, respectively. The maximum hydrogen production was achieved in the + 0.542 V reactor.

### Electrons transferred during the fermentation process

In order to guide the industrial application of electro-fermentation of xylose and facilitate precise control of the output, we analyzed the electron equivalents during the electro-fermentation process. Due to the complexity of the process, only the direct electro-fermentation of xylose to produce ethanol, acetate, and hydrogen was analyzed. The secondary oxidation process of the ethanol and acetate was neglected.

**Fig. 4:**
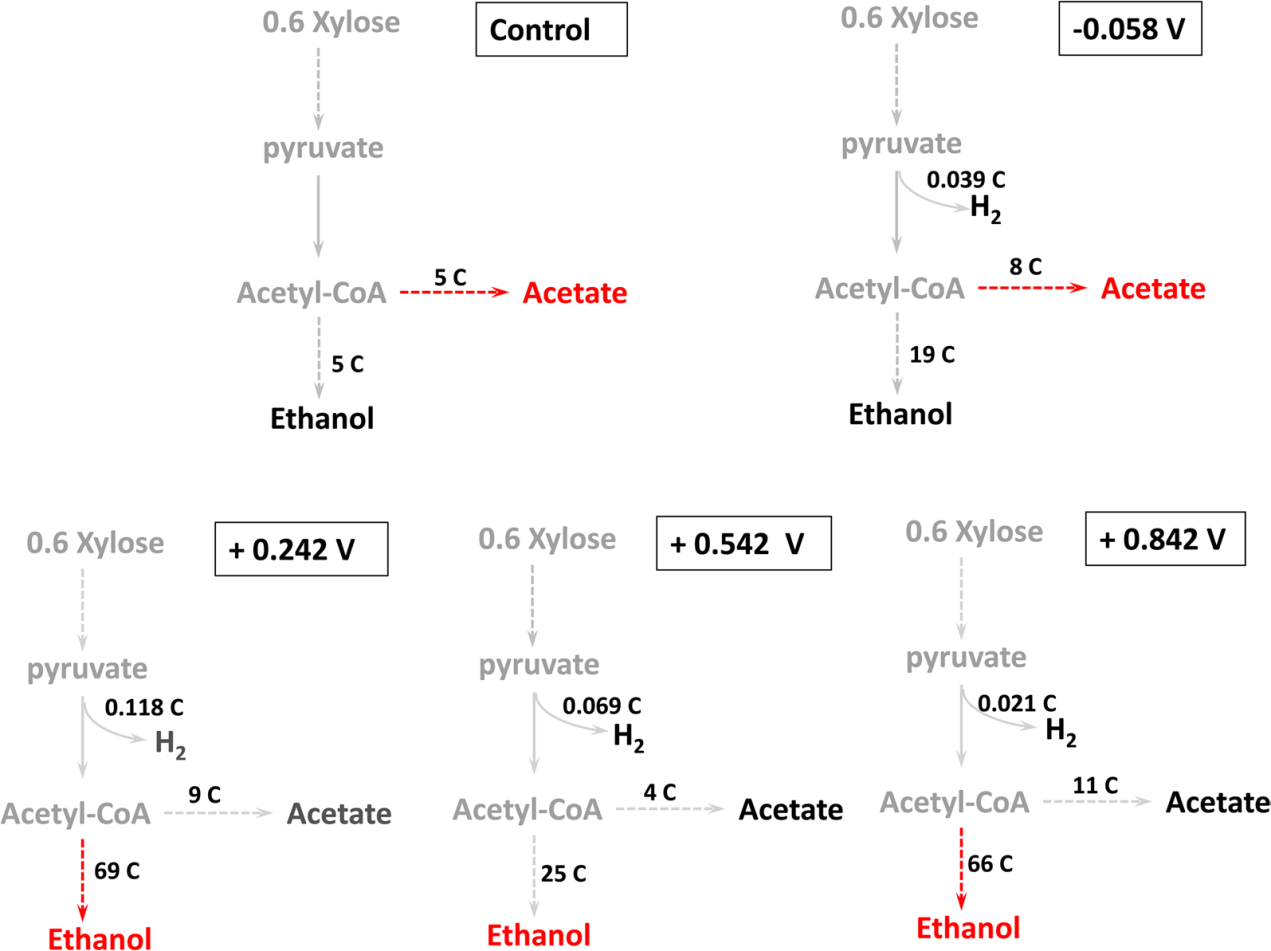
Overview of possible metabolism and electron transfer in reactors under different potential application conditions of − 0.058 V, + 0.242 V, + 0.542 V, and + 0.842 V vs. the standard hydrogen electrode

According to the Embden–Meyerhof–Parnas (EMP) pathway, 0.6 mol of xylose is converted to 1 mol of pyruvate. Further cleavage of 1 mol of pyruvate to 1 mol acetate yields 1 mol of hydrogen [11]. During fermentation, 0.6 mol of xylose produces 1 mol of ethanol. The distribution of transferred electron equivalents among the products was calculated according to Eqs. (1–2) and shown in Fig. 4.

1 $$0.{\text{6 C}}_{{5}} {\text{H}}_{{{1}0}} {\text{O}}_{{5}} \to {\text{ C}}_{{2}} {\text{H}}_{{5}} {\text{OH }} + {\text{ CO}}_{{2}}$$

2 $$0.{\text{6 C}}_{{5}} {\text{H}}_{{{1}0}} {\text{O}}_{{5}} + {\text{ H}}_{{2}} {\text{O }} \to {\text{ C}}_{{2}} {\text{H}}_{{4}} {\text{O}}_{{2}} + {\text{ CO}}_{{2}} + {\text{ 2H}}_{{2}}$$

The moles of electrons obtained from xylose consumption are calculated from the amount of the xylose consumed and converted to coulombs (${C}_{xy}$) using Eq. (3). At 150 h, the coulombs of ethanol obtained from the xylose consumption in the − 0.058, + 0.242 V, + 0.542 V, and + 0.842 V reactors were 19 C, 69 C, 25 C and 66 C, respectively. Ethanol in the + 0.242 V reactor took the largest product share of total amount of electrons. In summary, the + 0.242 V and + 0.842 V reactors achieved high electron equivalents transferred from xylose to the by-product ethanol. The coulombs of acetate obtained from the xylose consumption in the − 0.058 V, + 0.242 V, + 0.542 V, and + 0.842 V reactors were 8 C, 9 C, 4 C and 11 C, respectively. Acetate in the + 0.842 V reactor took the largest product share of total amount of electrons.

Coulombs consumed in the production of the measured H_2_ (${C}_{{H}_{2}}$) is estimated by calculating the moles of electrons consumed during the production of H_2_ and converting them to coulombs by Eq. (4). At 150 h, the coulombs consumed in the production of measured H_2_ in the − 0.058 V, + 0.242 V, + 0.542, and + 0.842 V reactors were 0.039 C, 0.118 C, 0.069 C and 0.021 C, respectively. We did not find the production of other gases such as methane in the reactor, suggesting that there might be no re-recycling of hydrogen.

Coulombs recovered as current intensity (${C}_{I}$) is calculated by integrating the current intensity from the initial to the final time of the batch experiment. At 150 h, the coulombs recovered as current intensity in the − 0.058 V, + 0.242 V, + 0.542 V, and + 0.842 V reactors by Eq. (5) were − 0.58 C, 0.17 C, 0.63 C, and 1.06 C, respectively.

Coulombic efficiency (CE) and cathodic gas recovery (*r*_*CAT*_) are usually as MEC performance indicators. The performance of a MEC is commonly assessed through the calculation of the CE using Eq. (6) and the ${r}_{CAT}$ using Eq. (7). CE compares the coulombs recovered as current intensity with the coulombs that could be theoretically generated from the substrate oxidation by anode microorganisms, while ${r}_{CAT}$ compares the coulombs consumed in produced H_2_ with the coulombs arriving to the cathode as current intensity. The CEs of the − 0.058 V, + 0.242 V, + 0.542 V, and + 0.842 V reactors were 4.38%, 0.35%, 3.57%, 2.3%, respectively. The ${r}_{CAT}$s of the − 0.058 V, + 0.242 V, + 0.542 V, and + 0.842 V reactors were 6.74%, 69.4%, 10.9%, and 2%, respectively.

In summary, among all the reactors, the + 0.842 V reactor has the best performance based upon its high production of ethanol.

### Potential application changes the fermentation mode

**Fig. 5:**
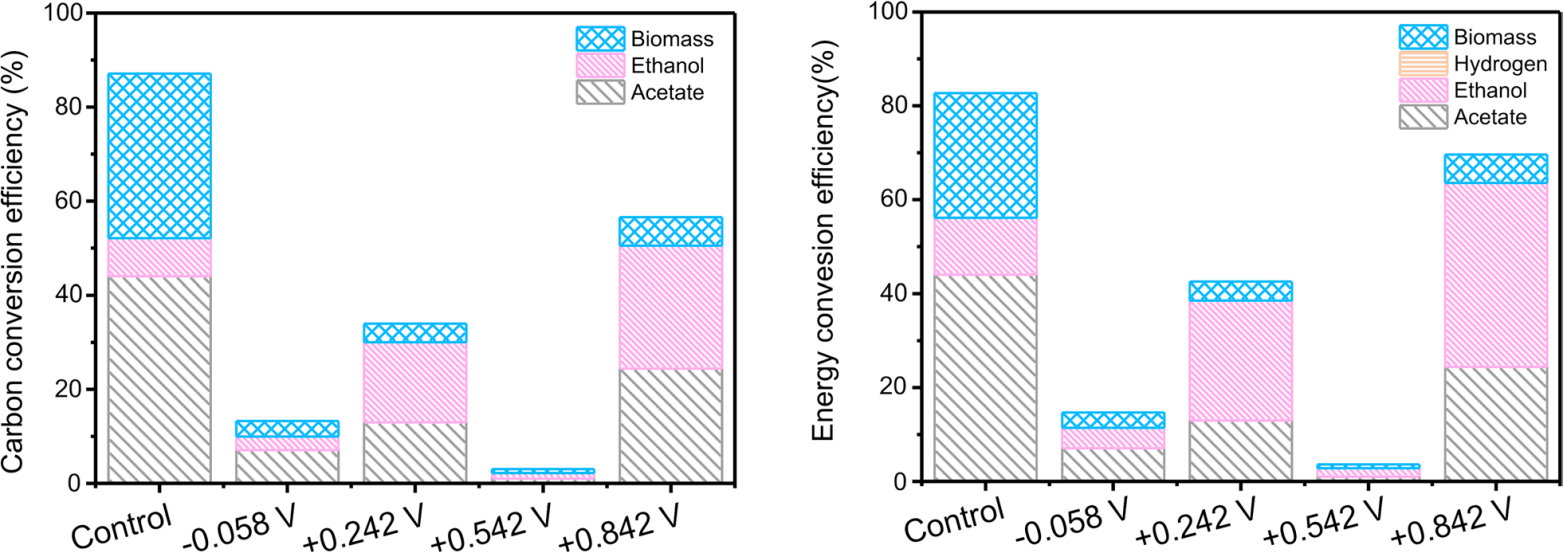
Carbon conversion efficiency and energy conversion efficiency in reactors under different potential application conditions of − 0.058 V, + 0.242 V, + 0.542 V, and + 0.842 V vs. the standard hydrogen electrode

Based on the quantification of substrate consumption, metabolite production, and cell yield, the profiles of the carbon and electron distribution in different batch cultures were analyzed (Fig. 5).

For control reactor, 79.1% of the carbon in the control reactor was converted into acetate (44.0%) and biomass (35.1%). Energy conversion efficiency (ECE) calculations also showed that acetate and biomass accounted for most of the energy. Electrons in this reactor mainly flowed to acetate production (44.0%) and biomass synthesis (26.6%), and a minor portion of electrons were maintained to ethanol (12.1%). This suggests that under the condition of no applied potential, the metabolism of activated sludge using xylose as the substrate is mainly for acid production and growth of microorganisms. A similar trend was also found in the − 0.058 V reactor.

However, applying positive potentials (+ 0.242 V, + 0.542 V, or + 0.842 V) made the microorganisms convert the fermentation mode for ethanol production. For the + 0.242 V reactor, most carbons remained in xylose. Only about 34.0% of the carbon was transferred into metabolic products and biomass. Ethanol (17.0%), acetate (13.0%) and biomass (4.0%) accounted for the most carbon by in the + 0.242 V reactor. The electrons also flowed to ethanol (25.6%), acetate (13.0%) and biomass (4.0%). For the + 0.542 V reactor, although CCE (3.0%) and ECE (3.6%) were both the lowest among all the reactors, fermentation mode also tended to ethanol fermentation. In the reactors with applied potentials, the highest carbon conversion efficiency (CCE) (56.6%) and ECE (69.6%) were obtained in the + 0.842 V reactor. The CCE in ethanol (26.1%) was higher than that of acetate (24.4%) in the + 0.842 V reactor. The electrons in the reactor flow to ethanol (39.1%) were higher than acetate (24.4%).

### Taxonomic assemblage of bacterial communities

Differences in output and electron flow of reactors in the ethanol, acetate and hydrogen production might be due to different microbial communities under different potentials. To identify the microorganisms that play a key role in various processes and reactors, the bacterial communities were extensively investigated at different stages by deep sequencing of the bacterial 16S rRNA gene amplicons.

**Fig. 6:**
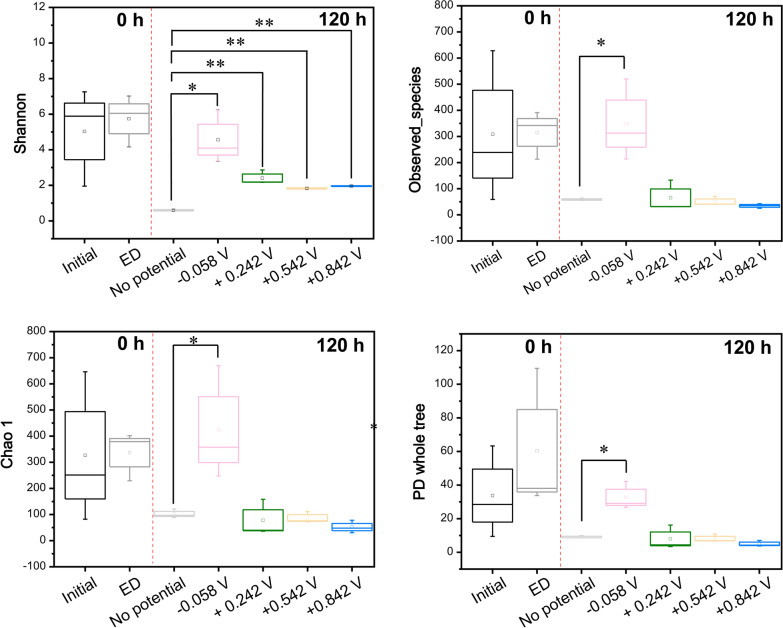
Changes of α-diversity indices. Boxplots of α-diversity indices, including the Shannon diversity index, Observed_species, and Chao1 index and PD whole tree. An analysis of variance was performed to test the significance of differences among zones. The symbol ‘□’ represents the average value; the horizontal line in the box represents the median value. Significant differences were analyzed compared with “No potential” in each diversity index sub-graph. Symbols *, ** indicate *P* < 0.05 and *P* < 0.01, respectively

Analysis of Shannon indices, observed species, Chao1 indices, PD whole tree revealed that there were no significant differences in different reactors (“Initial” and “ED”, respectively, in Fig. 6).

The diversity in the reactor without an applied potential had a Shannon index of 0.60 ± 0.06, Observed_ species of 59 ± 5, Chao1 index of 102.4 ± 13.8, and PD index of 9.1 ± 0.8 (Fig. 6). After potential application, the Shannon index varied significantly to 4.6 ± 1.5 (*P* < 0.05) with − 0.058 V application, 2.4 ± 0.4 (*P* < 0.01) with + 0.242 V application, 1.82 ± 0.06 (*P* < 0.01) with + 0.542 V application, 1.96 ± 0.04 (*P* < 0.01) with + 0.842 V application. The Observed_ species changed to 349 ± 156 (*P* < 0.05) with − 0.058 V application, 31.5 ± 0.7 (*P* > 0.05) with + 0.242 V application, 50 ± 18 (*P* > 0.05) with + 0.542 V application, 34 ± 9 (*P* > 0.05) with + 0.842 V application. The Chao1 index varied to 424.8 ± 178.5 (*P* < 0.05) with − 0.058 V application, 38 ± 2.6 (*P* > 0.05) with + 0.242 V application, 86.7 ± 18.2 (*P* > 0.05) with + 0.542 V application, 52.1 ± 19.8 (*P* > 0.05) with + 0.842 V application. The PD index varied to 32.7 ± 8.5 (*P* < 0.05) with − 0.058 V application, 8.0 ± 7.1 (*P* > 0.05) with + 0.242 V application, 8.1 ± 2.3 (*P* > 0.05) with + 0.542 V application, 5.0 ± 1.8 (*P* > 0.05) with + 0.842 V application. The bacterial α-diversity range was strongly influenced by the applied potentials in the reactors.

**Fig. 7:**
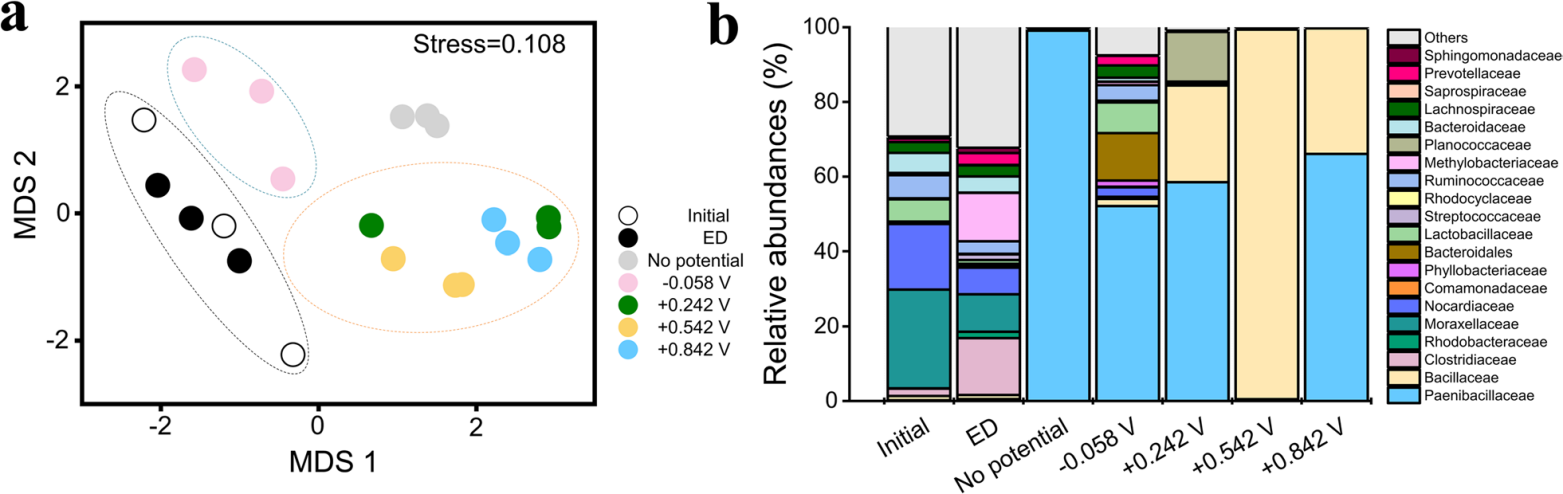
NMDS and relative abundances analysis of OTU in different reactors. **(a)** Comparison of the bacterial community structures using NMDS plots based on the Bray–Curtis dissimilarity. The NMDS plots display two time points (0 and 150 h) and four potentials (− 0.058, + 0.242, + 0.542, and + 0.842 V vs. the standard hydrogen electrode). The different symbols and colors indicate different reactors and applied potentials. (**b)** Relative abundances of the classified bacterial families. The relative abundance was based on the proportional frequencies of sequences that could be classified at a 97% similarity level. The relative abundances of different families were obtained by triplicate DNA extraction from each sample

In the non-metric multidimensional scaling (NMDS) plots, a separation in bacterial community composition was clearly observed between low potential (− 0.058 V) and higher potentials (+ 0.242 V, + 0.542 V, and + 0.842 V) at 150 h. This indicated that the applied potential significantly affected the bacterial community structure (Fig. 7a).

According to Adonis analysis, the differences in bacterial community composition between the group (no potential, − 0.058 V) and higher potentials application group (+ 0.242 V, + 0.542 V, and + 0.842 V) at 150 h were significant (*P* = 0.001 Table 1).

**Table 1:** The bacterial community differences with group (no potential, − 0.058 V) and higher potentials application group (+ 0.242 V, + 0.542 V and + 0.842 V) at 150 h by Adonis analysis

	R^2a^	*P*^a^
150 h^b^	0.473	0.001***^c^

^a^Statistic (R^2^) and significance (*P*) of differences between groups (with or without potential application for 150 h) are calculated by Adonis analysis using OTU-based Bray–Curtis distances

^b^150 h: samples with or without potential application for 150 h

^c^, *** is used to show statistical significance at the 0.001 level

### Community structure of the top 20 bacterial families in different stages

The dominant families were different between the initial sludge and domesticated sludge (ED) in the reactors. As shown in Fig. 7b, the major families in the initial samples included *Moraxellaceae* (26.43% ± 4.88%), *Nocardiaceae* (17.47% ± 2.34%), *Lactobacillaceae* (5.93% ± 2.60%), *Ruminococcaceae* (6.17% ± 2.3%), and *Bacteroidaceae* (5.33% ± 0.38%); they accounted for 61.33% of the bacterial sequences. In the ED samples, the major families were *Clostridiaceae* (15.2% ± 7.67%), *Moraxellaceae* (10.03% ± 4.14%), *Nocardiaceae* (7.13% ± 3.3%), and *Methylobacteriaceae* (12.97 ± 6.14%).

**Fig. 8:**
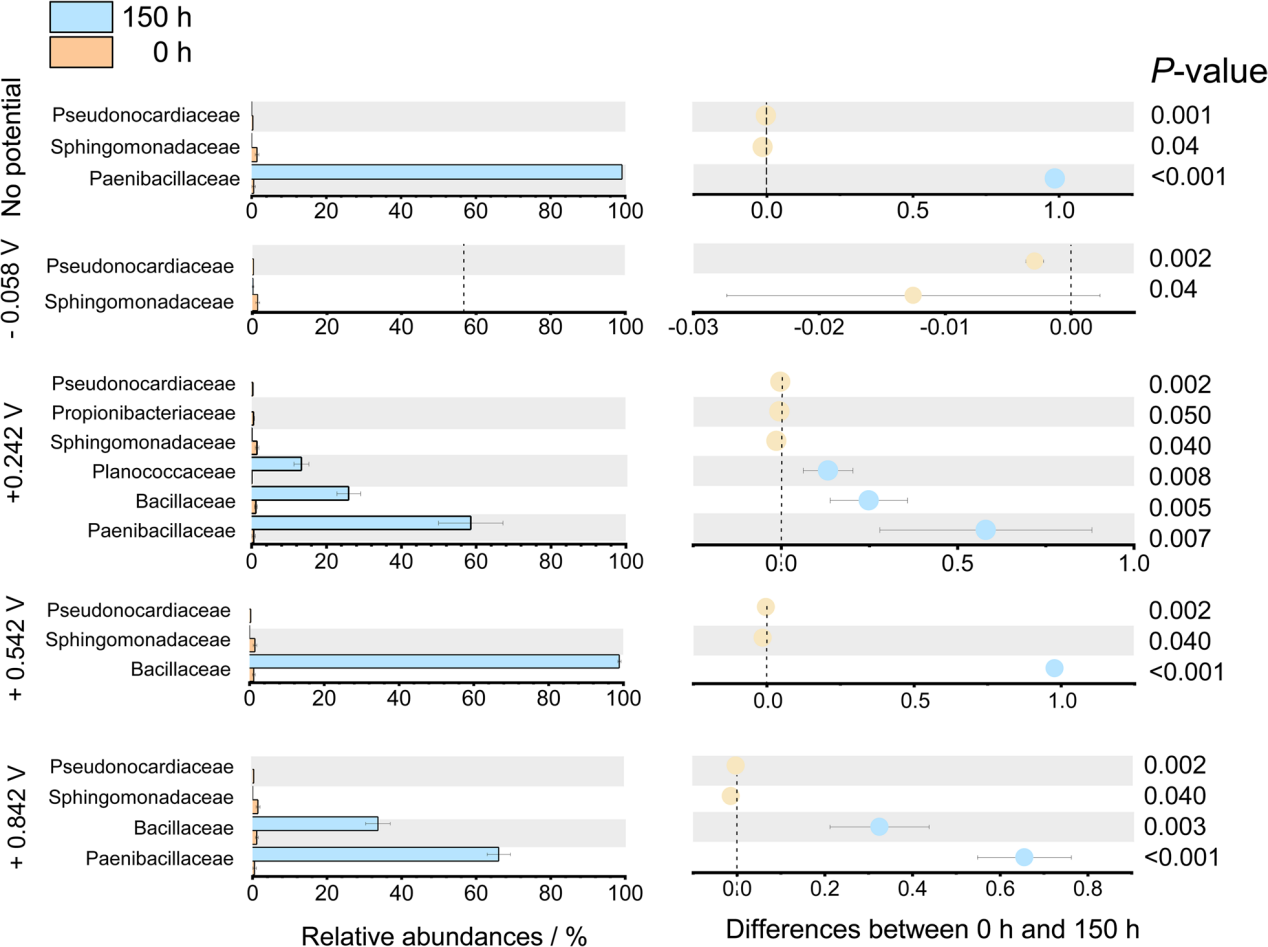
Significant differences of classified bacterial families between different reactors. The left panel shows differences of relative abundance in groups at 0 h and 150 h. The graph on the right shows the difference between groups according to the confidence level. The leftmost endpoint of each circle in the graph represents the lower 95% confidence interval of the mean difference, and the rightmost endpoint of the circle represents the upper 95% confidence interval. The center of the circle represents the difference in means. The group represented by the circle color has a high mean value. The far right displays the *P*-values for the significance test between the two time points of the corresponding family

After potential application, a shift in communities was clearly observed between the top 20 families. *Paenibacillaceae* and *Bacillaceae* were the two dominant families and accounted for 54.03–99.69% in almost all reactors (Fig. 7b). In the + 0.542 V reactor, *Bacillaceae* accounted for almost 100% of the relative abundance (Fig. 7b). T-test analysis between groups before and after potential application showed significant differences in the families (*P* < 0.05) (Fig. 8). *Paenibacillaceae* showed positive responses in + 0.242 V (*P* = 0.007) and + 0.842 V (*P* < 0.001) reactors. *Bacillaceae* presented positive responses in + 0.242 V (*P* = 0.005), + 0.542 V (*P* < 0.001), and + 0.842 V (*P* = 0.003) reactors. This suggests that the applied potential may help *Paenibacillaceae* and *Bacillaceae* growth and that they may utilize the electrons in the reactor.

While *Pseudonocardiaceae* and *Sphingomonadaceae* showed negative responses (*P* < 0.05) in all the reactors. *Propionibacteriaceae* (*P* < 0.05) showed negative responses in the + 0.242 V reactor (Fig. 8).

## Discussion

### Application of potential increases the xylose consumption and the ethanol production

Here we reported a MEC platform driven by active sludge with different applied potentials for the fermentation of xylose into ethanol. Xylose consumption and byproducts production (acetate and ethanol) were low without potential application. Among them, the highest xylose consumption rate is 92.4% observed in the + 0.542 V reactor. It was higher than 91% under 180℃ high temperature pyrolysis [12] and xylose consumption rate 80% by enzymatic hydrolysis after steam explosion [13]. Thus + 0.542 V potential is recommended for effective xylose fermentation in a MEC.

The + 0.842 V reactor achieved the maximal ethanol yield of 0.652 mol mol^−1^ xylose. It is lower than the 1.33 mol mol^−1^ xylose obtained from the batch fermentation of thermophilic anaerobic bacteria at 55 ℃ [14], but higher than the 0.47 mol mol^−1^ xylose obtained from the fermentation of mixed culture *Clostridium* sp. and *Klebsiella* sp. at 37 ℃ [15]. Thus, + 0.842 V potential is recommended for effective ethanol production in a MEC.

### Different potential changes the community structure of microorganisms and also the fermentation mode

The improvements in ethanol, acetate and hydrogen production by active sludge could be derived from the use of potential, resulting in reshaping the microbial communities, because each microbial community has its optimal potential [16]. The current peaks of applied potentials were different with time also reflect the continuous change of the microbial community with different potentials and time. NMDS and Adonis analysis indicates that the applied potentials changed the diversity and community structure of the dominant strains. The community structures in the reactors clearly differed between the low (− 0.058 V) and high potentials (+ 0.242 V, + 0.542 V, + 0.842 V). Meanwhile, the community structures in the reactor without applied electric potential and the reactor with negative electric potential (− 0.058 V) were also obviously different. This may be due to the fact that the optimal potentials of different microbial communities are different [16], so that the community structures have changed significantly during the process of potential changes. This also implies that different microbial structures in different reactors are in different metabolic process.

*Paenibacillaceae* and *Bacillaceae* were the two dominant families and accounted for 54.03–99.69% in almost all MEC reactors. They showed obvious (*P* < 0.05) positive responses. *Paenibacillaceae* has been reported to have a diverse set of carbohydrate-active enzymes and a complete pectin deconstruction system, so they can utilize xylose [17] for acid production [17, 18]. In no potential application reactor, *Paenibacillaceae* accounted for 99.1%. This indicates that *Paenibacillaceae* may metabolize xylose to produce ethanol and acetate, but may not produce hydrogen regardless of whether there is a potential application.

Further energy recovery analysis showed that the energy of the − 0.058 V reactor was mainly used for acetate production. High-throughput analysis showed that in addition to *Paenibacillaceae* that used xylose in the − 0.058 V reactor, *Bacteroidales* and *Lactobacillaceae* occupied a large proportion in the microbial communities. The current peaks at − 0.058 V may be due to the electron transfer between *Bacteroidales* and *Lactobacillaceae*. Different reports have shown that *Bacteroidales* is a relevant electron-donating strain in the microbial community of bioelectrochemical systems inoculated with wastewater or digestate [19–21]. In the − 0.058 V reactor, *Bacteroidales* presented a positive response indicated that its role in current production. This may be one of the reasons why the reactors changed from consuming electricity to producing electricity.

The energy recovery analysis showed that the energy in high potential of + 0.242 V, + 0.542 V, + 0.842 V reactors was mainly used to produce ethanol, and the highest ethanol energy recovery was obtained at + 0.842 V. This suggests that the application of negative potential makes *Paenibacillaceae* go through the acid-producing pathway, while the application of positive potential makes *Paenibacillaceae* go through the ethanol-producing pathway.

In the + 0.242 V, + 0.542 V, and + 0.842 V reactors, in addition to the positive response of *Paenibacillaceae*, *Bacillaceae*, another major family, was present in all reactors especially in the + 0.542 V reactor. Considering that *Bacillaceae* is able to produce hydrogen [22], and had a very large relative abundance in these reactors, we speculate that it may have produced hydrogen in these reactors. In addition to producing hydrogen, *Bacillaceae* are also involved in direct interspecies electron transfer [23]. The maximum peak current and the maximum proportion (99.4%) of *Bacillaceae* appeared in the + 0.542 V reactor at 150 h, which suggests that *Bacillaceae* may be able to transfer extracellular electrons to other microorganisms in the reactors. In the + 0.542 V reactor, the hydrogen output was higher than other reactors. *Bacillaceae* showed positive response at 150 h indicates that it might be the dominant hydrogen producer at + 0.542 V, although the production of hydrogen of all the reactors was relatively low. The reason for the lower hydrogen production may be the influence of ethanol. The production of hydrogen is catalyzed by NADH-dependent hydrogenase [24]. If NADH is consumed in the oxidative conversions of acetyl-CoA to ethanol, less NADH can be used for hydrogen production, which is also the reason for the lower hydrogen production [24]. Meanwhile, due to snatching NADH, higher hydrogen output may suppress ethanol and acid in + 0.542 V reactor. Thus, the production of ethanol and acid in + 0.542 V reactor was lower than other reactors. This suggests that the applied potential of + 0.542 V might cause the microorganisms to change from the xylose-degraded functional community to hydrogen and electricity generation community.

## Conclusion

The production of ethanol, acetate and hydrogen from xylose by active sludge was enhanced by applying potentials. Applied potentials changed the diversity and community structure of the dominant strains. Our work showed that different strategies could be used for the subsequent fermentation process with different needs. No applied potential can be used for acid production fermentation; − 0.058 V can be used to improve xylose consumption in the acid production process; + 0.242 V can be used for ethanol production fermentation and hydrogen production; + 0.542 V can be used to improve xylose consumption; + 0.842 V reactor has the best performance based upon its high production of ethanol. The energy recovery and carbon recovery efficiency of the + 0.842 V reactor is also the highest among the reactors with applied potential. Thus, + 0.842 V is best potential for energy-saving ethanol production.

## Materials and methods

### MFC/three-electrode system setup and operation

Single-chamber air-cathode MFCs were constructed by assembling a 28-mL single plastic chamber as previously reported with some modifications [25]. The distance between electrodes was 2 cm. Carbon cloth was used as the anode and had a projection area of 2 cm^2^. Commercial platinum catalyst (20 wt.% Pt/C, Alfa-Aesar) was utilized as catalyst of air-cathode. Air-cathode was processed by rolling method by using stainless steel mesh as current collector and the load of Pt was controlled to be about 0.5 mg cm^2^ [26]. Titanium wires were used to connect the anode and cathode to the external resistance. The reactor anode chambers were fed with 28 ml 50 mM phosphate buffer (4.09 g L^−1^ Na_2_HPO_4_, 3.32 g L^−1^ NaH_2_PO_4_·2H_2_O, 0.13 g L^−1^ KCl, 0.31 g L^−1^ NH_4_Cl) and 4 g L^−1^ xylose for about 6 days. When the voltage declined to below 0.001 V, 14 mL anodic liquid was replaced by 14 mL phosphate buffer with 4 g L^−1^ xylose. The external resistance was fixed at 1000 Ω, and all experiments were conducted at 30 °C.

Subsequently, 1 mL of the bacterial culture after acclimation with xylose in single-chamber air-cathode MFC was used as inoculum for each bioreactor with xylose as the substrate. A single-chamber membrane-less three-electrode system was constructed to investigate the ethanol production from the xylose-acclimated solution. The system was made from glass bottles (120 ml) with a working volumes of 100 ml. Carbon cloth (2 cm × 1 cm × 0.3 mm, projection area of 2 cm^2^), a platinum wire electrode (0.5 mm diameter, Aida, Tianjin, China), and a saturated calomel electrode were used as the working electrode, counter electrode, and reference electrode, respectively. For the electrolyte, 50 mM phosphate buffer saline was used. The electrodes and electrochemical workstation were connected with 0.8 mm titanium wires. The bottles were sealed tightly with silicone to avoid any gas leakage. During the testing, the working electrodes were kept at converted potentials − 0.058 V, + 0.242 V, + 0.542 V, or + 0.842 V vs. SHE. Each reactor was conducted with two replicates. Control experiments were conducted in reactors that were the same as the three-electrode system anaerobic reactors but without electrodes. The reactors were operated in batch mode, and digestion lasted for 150 h. All experiments were performed at 30 °C.

### Characterization of bioreactors

MFC reactors were characterized by the voltage (V) across an external resistor in the circuit, which were monitored at 1-min intervals with a Keithley 2700 data acquisition system (Tektronix, Beaverton, OR). Chronoamperometry measurements were used to confirm that electricity generation. Current–time (I–t) curves were plotted by electrochemical workstations (1030C, CH Instruments, Inc., China).

### Chemical analysis

The hydrogen in the reactor was drawn out by a syringe to measure the volume and components. The hydrogen was analyzed by gas chromatography (GC) 7820 (Agilent Technologies, USA) equipped with a thermal conductivity detector (TCD), as in the previous study [27]. The temperature of the column, temperature of the injector, and thermal conductivity were 80 °C, 250 °C, and 250 W m^−1^ °C^−1^, respectively.

The components and concentrations of the liquid samples were tested by high-performance liquid chromatography (HPLC) 1260 Infinity (Agilent Technologies, USA) equipped with a refractive index detector (RID). A Hi-plex H column (7.7 × 300 mm) was used to separate glucose and organic acids with an eluent of 5 mM H_2_SO_4_. The temperatures of the column and RID were 60 °C and 55 °C, respectively.

### Calculations

The degradation rate of substrate was calculated using the following equation:

3 $$\mathrm{Degradation\, rate \,of \,substrate}=\frac{\mathrm{ weight \,loss}(\mathrm{g})}{\mathrm{Initial \,substrate }(\mathrm{g})}\times 100\mathrm{\%}$$

Coulombs transferred from metabolites were calculated as in equation [28]:

4 $$C=F\cdot b\cdot V\cdot \Delta C\cdot {M}^{-1}$$

*F* is the Faraday constant (96,485 C mol^−1^ e^−^), *b* is the number of e transferred per mole of metabolites, *V* is the volume of liquid in the reactor. $\Delta C$ is the metabolites concentration change (g L^−1^), *M* is the molecular weight of metabolites (g mol^−1^). Total 3.4 e^−^ equiv for production of 1 mol ethanol from 0.6 mol xylose; Total 0.6 e^−^ equiv for production of 1 mol acetate from 0.6 mol xylose; Total 2e^−^ equiv for production of 1 mol H_2_ from 0.6 mol xylose.

Coulombs recovered as current were calculated as in Eq. (5) [28]:

5 $$C_{I} = \mathop \int \limits_{{t_{0} }}^{{t_{f} }} Idt$$

CE was calculated as in Eq. (6) [28]:

6 $$\mathrm{CE}=\frac{{\int }_{{t}_{0}}^{{t}_{f}}Idt}{F\cdot b\cdot V\cdot \Delta C\cdot {M}^{-1}}\times 100\mathrm{\%}$$

where *t*_*0*_ and *t*_*f*_ are the initial and final times of an experiment, $\Delta C$ is the xylose concentration change between *t*_*0*_ and *t*_*f*_ (g L^−1^), *M* is the molecular weight of xylose (150.13 g mol^−1^), *b* is the number of e transferred per mole of xylose (20 mol e^−^ mol xylose^−1^), *F* is the Faraday constant (96,485 C mol^−1^ e^−^), I is the current intensity and *V* is the volume of liquid in the reactor.

*r*_*CAT*_ was calculated as in Eq. (7) [28]:

7 $${r}_{CAT}=\frac{{V}_{{H}_{2}}\cdot 2\cdot F\cdot {V}_{m}^{-1}}{{\int }_{{t}_{0}}^{{t}_{f}}Idt{\cdot M}^{-1}}\times 100\mathrm{\%}$$

where ${V}_{{H}_{2}}$ is the final volume of H_2_ and *V*_*m*_ is the molar gas volume (24.03 L mol^−1^).

The CCE is defined as the ratio of the carbon content of metabolic products (eg., soluble metabolic products, biomass) to the total carbon of the xylose according to the modified Eq. 8 [29, 30]:

8 $${\text{CCE }} = \frac{{{\text{Carbon yield of ethanol}} + {\text{acetetate}} + {\text{biomass}}}}{{\text{Carbon yield of xylose consumed}}} \times 100{\text{\% }}$$

The ECE is defined as the ratio of the electron distribution among the fermentation products (e.g., ethanol, acetate, biomass and hydrogen) to the total number of electrons in the fermentation substrate according to the modified Eq. 9 [29, 30]:

9 $${\text{ECE }} = \frac{{{\text{Electron equivalents of Ethanol}} + {\text{Acetetate}} + {\text{H}}_{2} + {\text{biomass}}}}{{\text{Electron equivalents of Xylose}}} \times 100{\text{\% }}$$

The following correlations were used to calculate ECE: 1 mol xylose = 20 e^−^ equiv; 1 mol ethanol = 12 e^−^ equiv; 1 mol acetate = 8 e^−^ equiv; 1 mol H_2_ = 2e^−^ equiv; 1 mol biomass (C_5_H_7_O_2_N) = 20 e^−^ equiv. [31]

### Bacterial community analysis

To confirm the dominant microorganisms after batch experiments, microbial samples were obtained from each reactor for sequencing. DNA extractions, amplification and analysis were carried out by Novogene Co. Ltd. (Beijing, China). For polymerase chain reaction (PCR) amplification, a pair of universal 16S rRNA gene primers PS5 (341b4F-806R) was used for high-throughput sequencing [32]. The details of the microbial analysis were presented in a previous study [33, 34]. Briefly, DNA was extracted and amplified using the 16S V4 region primers (515F and 806R). Then amplicons were sequenced using the IonS5TMXL platform at Novogene Co. Ltd. (Beijing, China). Filtered sequences were classified as operational taxonomic unit (OTU) with 97% identity using the Uparse software (Uparse v7.0.1001, http://drive5.com/uparse/) [35]. Taxonomy was assigned to OTUs against SSUrRNA database of the SILVA (http://www.arb-silva.de/) [36]. The OTU table was rarefied to 63,933 sequences per sample in QIIME. Further data analysis was performed based on OTUs.

The microbial α-diversity was assessed using three metrics, including the Shannon index [37], the Observed_species, the Chao1 index [38], and PD whole tree.

### Nucleotide sequence deposition

All sequencing datasets were deposited in the National Center for Biotechnology Information (NCBI) Sequence Read Archive (http://trace.ncbi.nlm.nih.gov/Traces/sra/) with accession numbers SRR11087322-SRR11087363.

### Statistical analysis

NMDS analysis was performed with the R Vegan package. T-test was used to test R software to analyze the difference of β-diversity index between groups. The contribution of the applied potentials to the community composition was calculated separately through a permutational multivariate analysis of variance (PERMANOVA) with the ‘Adonis’ function in the vegan R package with 9999 random permutations. All statistical analyses were performed with Origin v8.1 software (OriginLab Corp., Northampton, MA, USA) and R software v3.4.4 (https://www.r-project.org) (Additional file 1: Fig. S1).

## Supplementary Information

**Additional file 1: Figure S1.** Influence of potential application on cell growth and the pH values in fermentative cultures. * is used to show statistical significance at the 0.05 levels.

## Data Availability

The datasets used and/or analyzed during the current study are available from the corresponding author on reasonable request.

## Abbreviations

MFCs: Microbial fuel cells
EET: Extracellular electron transfer
EMP: Embden–Meyerhof–Parnas
CE: Coulombic efficiency
${r}_{CAT}$: Cathodic gas recovery
ECE: Energy conversion efficiency
CCE: Carbon conversion efficiency
NMDS: Non-metric multidimensional scaling
SHE: Standard hydrogen electrode
GC: Gas chromatography
TCD: Thermal conductivity detector
HPLC: High-performance liquid chromatography
RID: Refractive index detector
PCR: Polymerase chain reaction
PERMANOVA: Permutational multivariate analysis of variance

## References

[1] Boboescu IZ, Gherman VD, Lakatos G, Pap B, Biro T, Maroti G (2016). Surpassing the current limitations of biohydrogen production systems: The case for a novel hybrid approach. Bioresour Technol.

[2] Mielenz JR (2001). Ethanol production from biomass: technology and commercialization status. Curr Opin Microbiol.

[3] Sivers MV, Zacchi G, Olsson L, Hahn-Haegerdal B (2010). Cost analysis of ethanol production from willow using recombinant *Escherichia coli*. Biotechnol Progr.

[4] Wiselogel A, Tyson S, Johnson D (1996). Biomass feedstock resources and composition. Fuel Energ Abstr.

[5] Bothast RJ, Nichols NN, Dien BS (1999). Fermentations with new recombinant organisms. Biotechnol Prog.

[6] Olofsson K, Bertilsson M, Liden G (2008). A short review on SSF—an interesting process option for ethanol production from lignocellulosic feedstocks. Biotechnol Biofuels.

[7] Dionisi D, Anderson JA, Aulenta F, Mccue A, Paton G (2015). The potential of microbial processes for lignocellulosic biomass conversion to ethanol: a review. J Chem Technol Biot.

[8] Ester M (2015). Barbara, Scaglia, Andrea, Schievano, Fabrizio, Adani: dark fermentation effectiveness as a key step for waste biomass refineries: influence of organic matter macromolecular composition and bioavailability. Int J Energ Res.

[9] Rago L, Pant D, Schievano A. Electro-fermentation—microbial electrochemistry as new frontier in biomass refineries and industrial fermentations. Advanced Bioprocessing for Alternative Fuels, Biobased Chemicals, and Bioproducts. Woodhead publishing, 2019; pp. 265–87

[10] Agler MT, Wrenn BA, Zinder SH, Angenent LT (2011). Waste to bioproduct conversion with undefined mixed cultures: the carboxylate platform. Trends Biotechnol.

[11] Taylor MP, Eley KL, Martin S, Tuffin MI, Burton SG, Cowan DA (2009). Thermophilic ethanologenesis: future prospects for second-generation bioethanol production. Trends Biotechnol.

[12] Xu YR, Wang PF, Xue SW, Kong FG, Ren H, Zhai HM (2020). Green biorefinery - the ultra-high hydrolysis rate and behavior of Populus tomentosa hemicellulose autohydrolysis under moderate subcritical water conditions. Rsc Adv.

[13] Zheng J, Choo K, Bradt C, Lehoux R, Rehmann L (2014). Enzymatic hydrolysis of steam exploded corncob residues after pretreatment in a twin-screw extruder. Biotechnol Rep.

[14] Silva V, Ratti RP, Sakamoto IK, Andrade MVF, Varesche MBA (2018). Biotechnological products in batch reactors obtained from cellulose, glucose and xylose using thermophilic anaerobic consortium. Renew Energ.

[15] Maintinguer SI, Fernandes BS, Duarte ICS, Saavedra NK, Adorno MAT, Varesche MBA (2011). Fermentative hydrogen production with xylose by Clostridium and Klebsiella species in anaerobic batch reactors. Int J Hydrogen Energ.

[16] Logan BE (2008). Microbial fuel cells.

[17] Sanchez MM, Fritze D, Blanco A, Sproer C, Tindall BJ, Schumann P, Kroppenstedt RM, Diaz P, Pastor FI (2005). *Paenibacillus barcinonensis* sp. nov., a xylanase-producing bacterium isolated from a rice field in the Ebro River delta. Int J Syst Evol Microbiol.

[18] Shida O, Takagi H, Kadowaki K, Nakamura LK, Komagata K (1997). Emended description of *Paenibacillus amylolyticus* and description of *Paenibacillus illinoisensis* sp. nov. and *Paenibacillus chibensis* sp. nov.. Int J Syst Bacteriol.

[19] Croese E, Pereira MA, Euverink GJ, Stams AJ, Geelhoed JS (2011). Analysis of the microbial community of the biocathode of a hydrogen-producing microbial electrolysis cell. Appl Microbiol Biotechnol.

[20] Daghio M, Gandolfi I, Bestetti G, Franzetti A, Guerrini E, Cristiani P (2015). Anodic and cathodic microbial communities in single chamber microbial fuel cells. Nat Biotechnol.

[21] Malvankar NS, Lau J, Nevin KP, Franks AE, Tuominen MT, Lovley DR (2012). Electrical conductivity in a mixed-species biofilm. Appl Environ Microbiol.

[22] Nandi R, Sengupta S (1998). Microbial production of hydrogen: an overview. Crit Rev Microbiol.

[23] Zhuang L, Tang J, Wang Y, Hu M, Zhou S (2015). Conductive iron oxide minerals accelerate syntrophic cooperation in methanogenic benzoate degradation. J Hazard Mater.

[24] Schmitz O, Bothe H (2010). NAD(P)+-dependent hydrogenase activity in extracts from the cyanobacterium *Anacystis nidulans*. FEMs Microbiol Lett.

[25] Gao C, Wang A, Wu W, Yin Y, Zhao Y (2014). Enrichment of anodic biofilm inoculated with anaerobic or aerobic sludge in single chambered air-cathode microbial fuel cells. Bioresource technol.

[26] Liu Q, Chen S, Zhou Y, Zheng S, Hou H, Zhao F (2014). Phosphorus-doped carbon derived from cellulose phosphate as efficient catalyst for air-cathode in microbial fuel cells. J Power Sources.

[27] Montpart N, Rago L, Baeza JA, Guisasola A (2015). Hydrogen production in single chamber microbial electrolysis cells with different complex substrates. Water Res.

[28] Ruiz Y, Baeza JA, Guisasola A (2013). Revealing the proliferation of hydrogen scavengers in a single-chamber microbial electrolysis cell using electron balances. Int J Hydrogen Energ.

[29] Cheng J, Ding L, Lin R, Yue L, Liu J, Zhou J, Cen K (2016). Fermentative biohydrogen and biomethane co-production from mixture of food waste and sewage sludge: effects of physiochemical properties and mix ratios on fermentation performance. Appl Energ.

[30] Fangkum A, Reungsang A (2011). Biohydrogen production from sugarcane bagasse hydrolysate by elephant dung: effects of initial pH and substrate concentration. Int J Hydrogen Energ.

[31] Rittmann BE, Mccarty PL (2001). Environmental biotechnology : principles and applications.

[32] Lu YZ, Ding ZW, Ding J, Fu L, Zeng RJ (2015). Design and evaluation of universal 16S rRNA gene primers for high-throughput sequencing to simultaneously detect DAMO microbes and anammox bacteria. Water Res.

[33] Chen L, Zhang P, Shang W, Zhang H, Li Y, Zhang W, Zhang Z, Liu F (2018). Enrichment culture of electroactive microorganisms with high magnetic susceptibility enhances the performance of microbial fuel cells. Bioelectrochemistry.

[34] Wang M, Chen L, Li Y, Chen L, Liu Z, Wang X, Yan P, Qin S (2018). Responses of soil microbial communities to a short-term application of seaweed fertilizer revealed by deep amplicon sequencing. Appl soil ecol.

[35] Edgar RC (2013). UPARSE: highly accurate OTU sequences from microbial amplicon reads. Nat Methods.

[36] Quast C, Pruesse E, Yilmaz P, Gerken J, Schweer T, Yarza P, Peplies J, Glockner FO (2012). The SILVA ribosomal RNA gene database project: improved data processing and web-based tools. Nucleic Acids Res.

[37] Hill TC, Walsh KA, Harris JA, Moffett BF (2003). Using ecological diversity measures with bacterial communities. FEMS microbiol ecol.

[38] Chao A, Bunge J (2002). Estimating the number of species in a stochastic abundance model. Biometrics.

