# Supplementary figures and images for: Application of electric potential improves ethanol production from xylose by active sludge
Source: Biotechnol Biofuels. 2021 Nov 17;14:215. doi: 10.1186/s13068-021-02065-y (PMC8596957; doi:10.1186/s13068-021-02065-y)

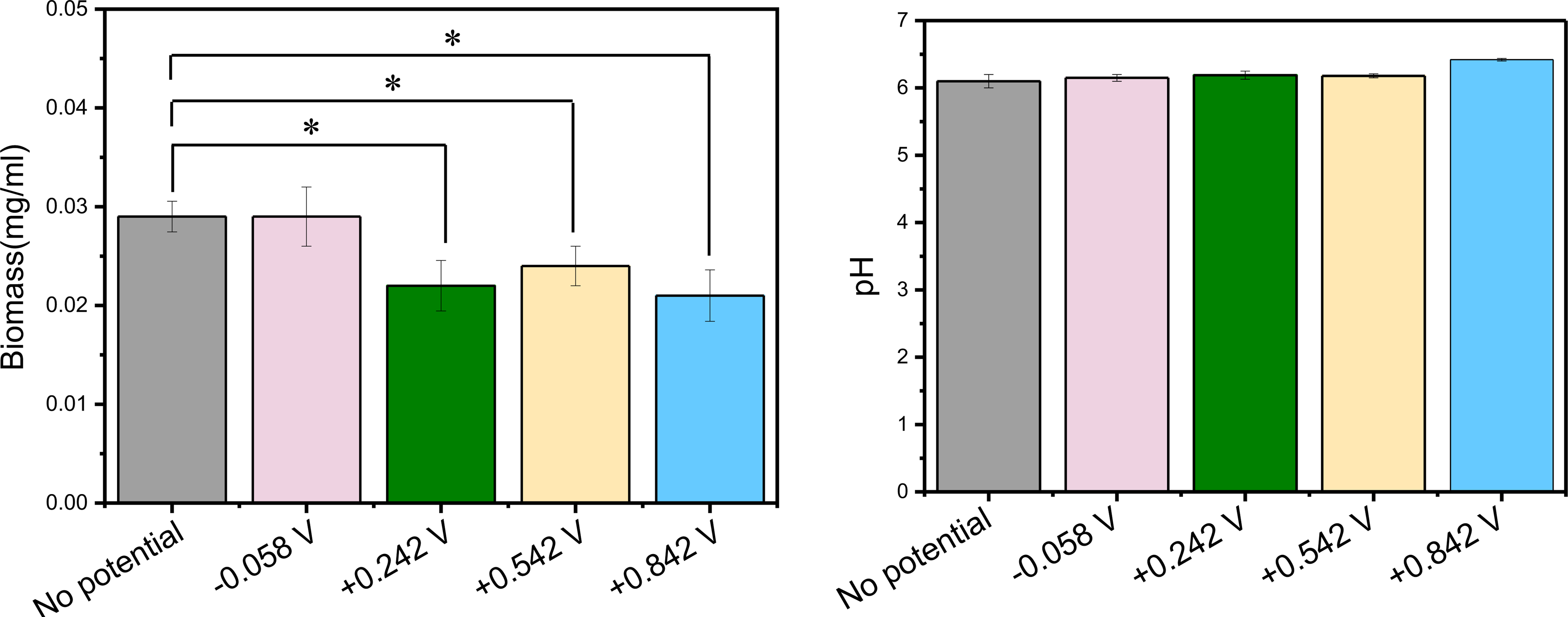

Supplement: Supplementary file 1 — Additional file 1: Figure S1. Influence of potential application on cell growth and the pH values in fermentative cultures. * is used to show statistical significance at the 0.05 levels. [file 13068_2021_2065_MOESM1_ESM.tif]
